# LncRNAMORT is upregulated in myocardial infarction and promotes the apoptosis of cardiomyocyte by downregulating miR-93

**DOI:** 10.1186/s12872-020-01522-0

**Published:** 2020-05-25

**Authors:** Jing Lv, Yi Zhu, Shanglong Yao

**Affiliations:** Department of Anesthesiology, Institute of Anesthesiology and Critical Care Medicine, Union Hospital, Tongji Medical College, Huazhong University of Science and Technology, No.1277 Jiefang Avenue, Wuhan City, Hubei Province 430000 People’s Republic of China

**Keywords:** Myocardial infarction, lncRNA MORT, miR-93, Cardiomyocyte, Apoptosis

## Abstract

**Background:**

Myocardial infarction (MI) affects the expression of a large number of lncRNAs, while the functions of those dysregulated lncRNAs are mostly unclear.

**Materials and methods:**

Expression of MORT and miR-93 in hearth tissues and plasma of both MI mice and Sham mice and both MI patients and healthy controls was detected by RT-qPCR. Correlations of expression levels of MORT and miR-93 between hear tissues and plasma of MI mice were explored by performing linear regression.

**Results:**

In the present study we found that MORT expression levels were higher, while expression levels of miR-93 were lower in both plasma and heart tissues of mice MI mice models compared with Sham mice. Plasma levels of MORT and miR-93 were largely consistent with expression levels of MORT and miR-93 in heart tissue of MI mice. MORT expression levels were also higher, while levels of miR-93 were also lower in plasma of MI patients compared with healthy controls. MORT and miR-93 were inversely correlated in MI patients but not in healthy controls. MORT overexpression resulted in inhibited miR-93 expression in cardiomyocytes (AC16 cell line), while miR-93 overexpression did not significantly affect MORT expression. MORT overexpression promoted cardiomyocyte apoptosis, while miR-93 overexpression played and opposite role and attenuated the effects of MORT overexpression.

**Conclusion:**

Therefore, lncRNA MORT is upregulated in myocardial infarction and promotes the apoptosis of cardiomyocyte by downregulating miR-93.

## Background

Long non-coding RNAs (lncRNAs), is a group of RNA transcripts without protein-coding capacity or significant open reading frames [1, 2]. In spite of the lack of protein products, lncRNAs participate in both physiological and pathological processes through the regulation of downstream genes at multiple levels, such as methylation, posttranscriptional regulation and translational regulation [3, 4]. Both clinical and experimental experiments have revealed that lncRNAs are critical determinants in human diseases [5], and the regulation of lncRNAs expression may contribute to disease treatment [6]. However, most lncRNA studies focused on certain types of disease, such as cancer [7]. The function of lncRNAs in other diseases is largely unknown.

Myocardial infarction (MI) is one of the most serious ischemic heart diseases and is the leading cause of cardiovascular morbidity and mortality [8]. MI affects more than 50 million people worldwide. How to prevent and improve treatment of MI is a major task for public health research [9]. MI is affected by gender, age, atherosclerosis, arterial hypertension, dyslipidemia, diabetes, smoking and many other factors [10, 11]. It is also observed that abnormal expression of certain lncRNAs may result in MI pathogenesis [12]. For instance, lncRNA MIAT expression is altered in MI and it participates in almost all aspects of the progression of MI [12]. In effect, regulation of the expression of crucial lncRNA players in MI provides novel insights to the development of preventative and therapeutic approaches [13]. LncRNA MORT was recently characterized as a downregulated lncRNA in different types of cancer [14]. Preliminary microarray data showed that MORT was upregulated in MI mice (data not shown), and is inversely correlated with miR-93, which with protective effects on MI [15]. In the present study we explore the involvement of lncRNA MORT in MI and explore its possible interactions with miR-93.

## Methods

### Animal model

A total of 30 male Balb/C mice (18- 20 g, 8 to 12 weeks) were bought from Beijing Weitong Lihua Experimental Animal Technology Co., Lt. The mice were randomly divided into MI group and Sham groups, 15 in each group. MI model was established using the methods described by Kolk et al. [16]. Mice were anesthetized in an induction chamber with isoflurane (2%) before model construction. Mice in MI group and Sham groups went through the same procedure except that ligation of the left anterior descending artery was not performed for mice in Sham group. Mice were sacrificed by CO_2_ asphyxiation, and blood and heart tissues were collected. Blood was used to prepare plasma samples according to conventional method. In MI group but not in Sham group, Off-white colour was observed on the apex of the heart and myocardium located on the left ventricle anterior wall (data not shown), indicating the successful establishment of MI models in MI group [17]. Plasma levels of LDL and HDL were also significantly lower in MI group in comparison to Sham group, which supported the establishment of MI models in MI group. Heart tissues were collected from the infracted regions. Animal experiments and all experimental protocols were approved by Ethics Committee of Union Hospital, Tongji Medical College, Huazhong University of Science and Technology. All experiments were performed in accordance with national and institutional guidelines and regulations.

### Patients and plasma

A total of 56 patients with MI were enrolled in Union Hospital, Tongji Medical College, Huazhong University of Science and Technology from January 2016 to January 2018 to serve as research subjects. MI patents were diagnosed by electrocardiogram and blood tests for heart muscle cell damage. Inclusion criteria: 1) MI patients diagnosed for the first time; 2) no therapies were initiated before admission. Exclusion criteria: 1) patients complicated with multiple diseases; 2) patients transferred from other hospitals or had been treated before admission. The 56 MI patients included 29 males and 27 females, and age ranged from 43 to 68 years, with a mean age of 54.3 ± 6.6 years. During the same time period, a total of 46 healthy controls were also enrolled at the physical health center of Union Hospital, Tongji Medical College, Huazhong University of Science and Technology to serve as control group. Control group included 25 males and 21 females, and age ranged from 42 to 66 years, with a mean age of 54.8 ± 6.9 years. This study had been approved by the Ethics Committee of Union Hospital, Tongji Medical College, Huazhong University of Science and Technology. All participants signed informed consent. See Table 1 for clinical data of MI patients and healthy controls.

**Table 1:** Clinical data of MI patients and healthy controls

	MI (*n* = 56)	Control (*n* = 46)
Age (years)	54.3 ± 6.6	54.8 ± 6.9
Gender		
Male	29	25
Female	27	21
LVEF (%)	54.28 ± 4.91	65.18 ± 4.53
LDL (mg/dL)	100.23 ± 7.89	140.28 ± 9.91
HDL (mg/dL)	42.88 ± 4.52	52.29 ± 5.56

### Total RNA extractions and real-time quantitative reverse transcription PCR (qRT-PCR)

To detect the expression of lncRNA MORT RNAzol reagent (Sigma-Aldrich, St. Louis, MO, USA) was used to extract total RNA from heart tissue bordering the MI was well as in vitro cultivated cells. SuperScript III Reverse Transcriptase (Thermo Fisher Scientific) was used to perform reverse transcription and Applied Biosystems™ Power™ SYBR™ Green Master Mix was used to prepare all PCR reaction systems. To detect the expression of miR-93, miRNAs were extracted using miRNA Isolation Kit (RMI050, Geneaid), miRNA reverse transcription was performed using miScript II RT Kit (QIAGEN) and PCR reaction systems were prepared using miScript SYBR Green PCR Kit (QIAGEN). All PCR reactions were performed on CFX96 Touch Deep Well™ Real-Time PCR Detection System (Bio-Rad) with 18S RNA as the endogenous control of MORT and U6 as the endogenous control of miR-63. Primer sequences were: 5′-GGATCAGACTGATGATCACCAAC-3′ (forward) and 5′-ATGAAGGATTCATTGAATGCTGC-3′ (reverse) for MORT; 5′-CTACCACATCCAAGGAAGCA-3′ (forward) and 5′-TTTTCGTCACTACCTCCCCG-3′ (reverse) for 18S rRNA. Forward primer of 18S rRNA was 5′-CAAAGTGCTGTTCGTGCAGG-3′. Reverse primer of miR-93 and U6 primers were from the kit.

### Cell line

AC16 human cardiomyocyte cell line was used in this study to perform all in vitro cell experiments. Cells were cultivated in DMEM supplemented with 12% fetal bovine serum, 1% streptomycin and penicillin in an incubator (37 °C, 5% CO_2_).

### Vectors and cell transfection

Vectors (pcDNA3.1) expressing MORT (accession: NR_036521.1) were constructed by Sangon (Shanghai, China). MISSION® microRNA Mimic hsa-miR-93 and negative control miRNA were bought from Sigma-Aldrich. MORT siRNA and negative control siRNA were from Sangon (Shanghai, China). Lipofectamine 2000 Transfection Reagent (Thermo Fisher Scientific) was use to perform all cell transfections in strict accordance with manufacturer’s instructions. Un-transfected cells were control cells. Cells transfected with empty vectors, negative control miRNAs or negative control siRNA were negative control cells. Cells were harvested at 36 h after transfection for subsequent experiments.

### Cell apoptosis assay

Cells were harvested at 36 h after transfection to perform cell apoptosis assay. Briefly, single cell suspensions were prepared and cell density was adjusted to 3 × 10^4^ cells per ml. Cell suspensions were transferred to a 6-well plate with 2 ml in each well. Cells were cultivated (95% N2, 5% CO2) for 12 h, followed by 0.25% trypsin digestion. After that, propidium iodide (PI) and Annexin V-FITC (Dojindo, Japan) staining was performed and flow cytometry was carried out to detect apoptotic cells.

### Statistical analysis

All experiments were performed in triplicate manner and data were recorded as mean ± standard deviation. Comparisons between Sham and MI model mice, and between MI patients and healthy controls were performed by unpaired t test. Comparisons among multiple groups were performed by one-way ANOVA followed by Tukey test. Correlations between expression levels of MORT and miR-93 were performed by linear regression. All statistical analyses were performed using Graphpad Prism 6 software. Differences with *p* < 0.05 were statistically significant.

## Results

### MORT and miR-93 expression was altered in mice MI models

**Fig. 1:**
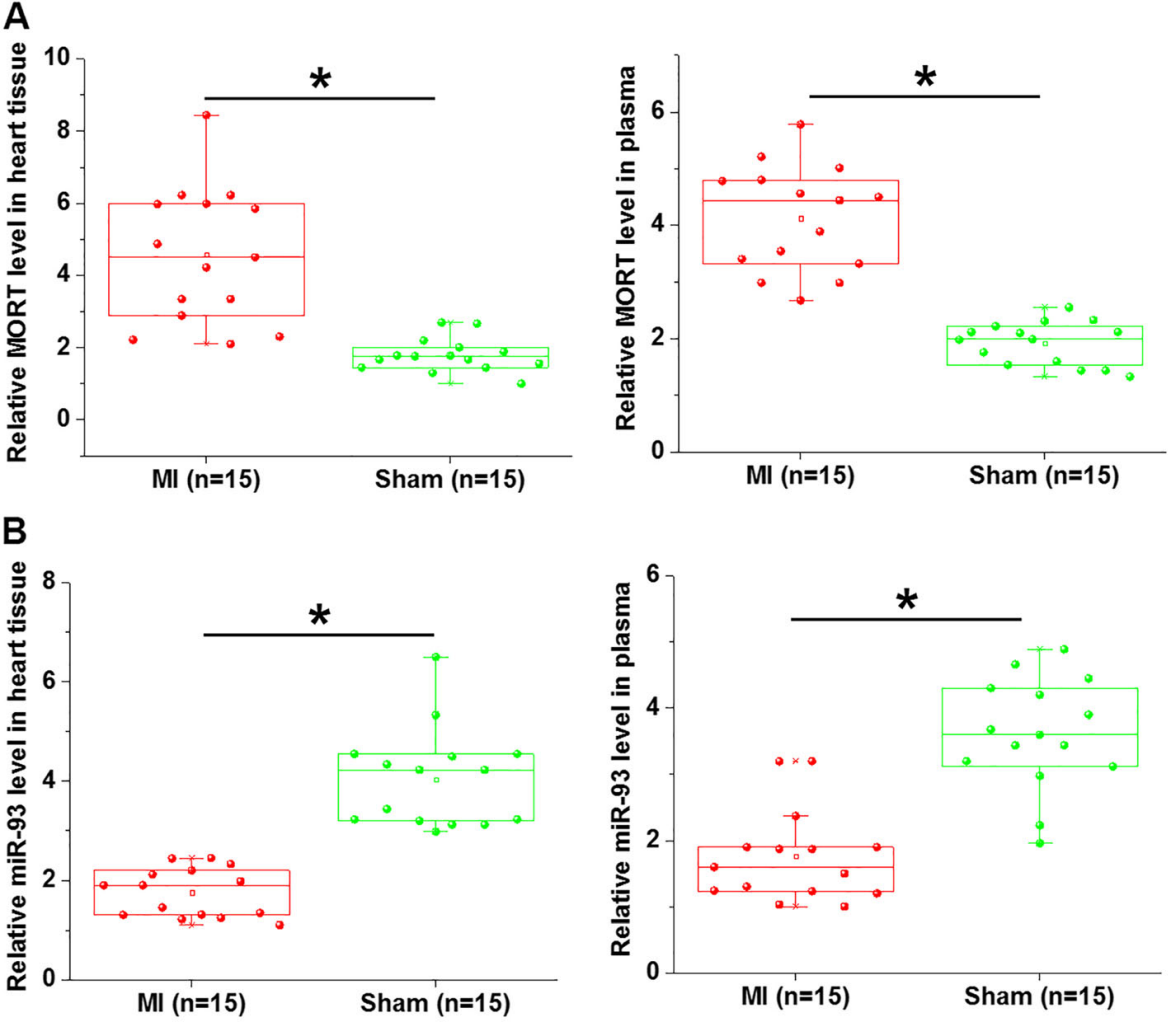
MORT and miR-93 expression was altered in mice MI models. RT-qPCR was performed to analyse the levels of MORT and miR-93 in both plasma and heart tissues from mice MI mice and Sham mice. Results of RT-qPCR showed that MORT expression levels were higher (A), while expression levels of miR-93 were lower (B) in both plasma and heart tissues of mice MI mice models compared with Sham mice (*, *p* < 0.05)

Expression of MORT and miR-93 in heart tissues and plasma of both MI mice and Sham mice was detected by RT-qPCR. Compared with Sham mice, MORT was significantly upregulated in both hearth tissues and plasma of MI model mice (Fig. 1a, *p* < 0.05). In contrast, miR-93 was significantly downregulated in both hearth tissues and plasma of MI model mice compared with Sham mice (Fig. 1b, *p* < 0.05).

### Plasma levels of MORT and miR-93 were largely consistent with expression levels of MORT and miR-93 in heart tissue in MI mice

**Fig. 2:**
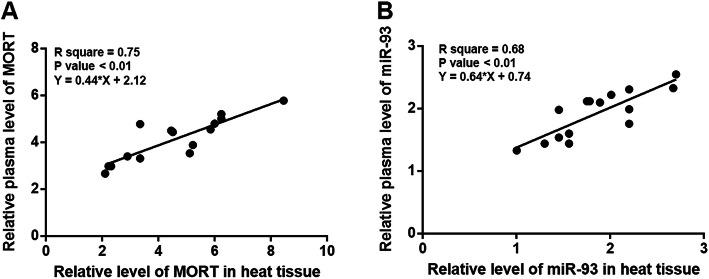
Plasma levels of MORT and miR-93 were largely consistent with expression levels of MORT and miR-93 in heart tissue in MI mice. Correlations between plasma levels of MORT and miR-93 and their levels in heart tissues were analysed by linear regression analysis. It was observed that plasma levels of MORT (**a**) and miR-93 (**b**) were significantly and positively correlated with expression levels of MORT and miR-93 in heart tissue in MI mice

Correlations of expression levels of MORT and miR-93 between hear tissues and plasma of MI mice were explored by performing linear regression. It was observed that expression of MORT in heart tissues and plasma were significantly and positively correlated (Fig. 2a). In addition, a positive correlation between expression levels of miR-93 in heart tissues and plasma was also observed (Fig. 2b).

### Plasma MORT and of miR-93 expression was also altered in MI patients

**Fig. 3:**
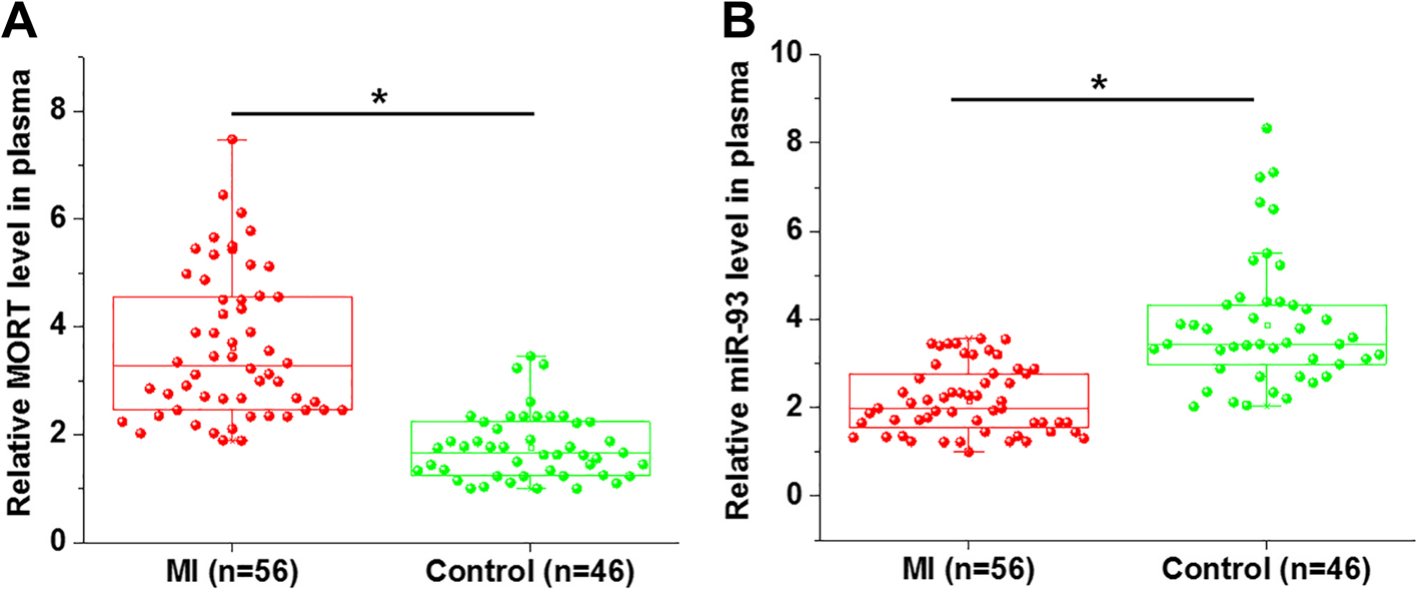
Plasma MORT and of miR-93 expression was also altered in MI patients. RT-qPCR was performed to analyse the levels of MORT and miR-93 in plasma from both MI patients and healthy controls. RT-qPCR results showed that MORT expression levels were significantly higher (**a**), while levels of miR-93 were significantly lower (**b**) in plasma of MI patients (*, *p* < 0.05)

Plasma levels of MORT and of miR-93 in MI patients and healthy controls were also measured by RT-qPCR. Compared with heathy controls, MORT expression levels were significantly higher (Fig. 3a), while levels of miR-93 were significantly lower (Fig. 3b) in plasma of MI patients (*p* < 0.05).

### MORT and miR-93 were inversely correlated in MI patients

**Fig. 4:**
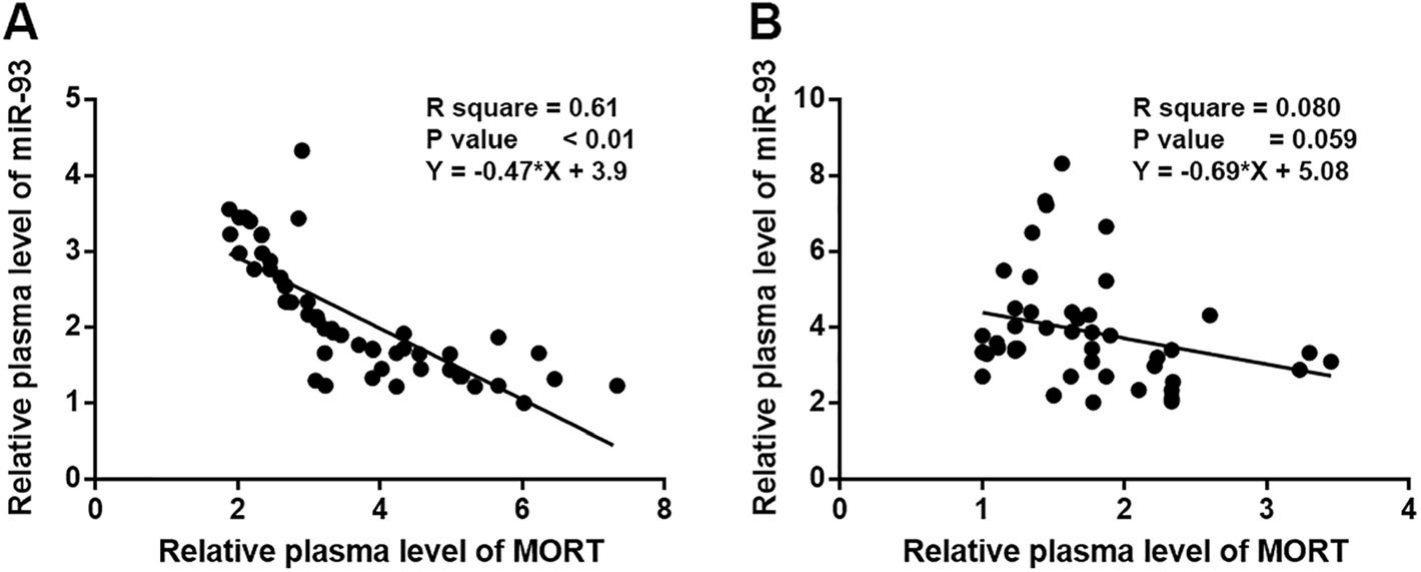
MORT and miR-93 were inversely correlated in MI patients. Correlations between plasma levels of MORT and miR-93 were analysed by linear regression. The analysis revealed a significant and inverse correlation between plasma levels of MORT and miR-93 in MI patients (**a**), but not in healthy controls (**b**)

Correlations between expression levels of MORT and miR-93 were performed by linear regression. The results showed a significant and inverse correlation between plasma levels of MORT and miR-93 in MI patients (Fig. 4a). However, the correlation between plasma levels of MORT and miR-93 was not significant in healthy controls (Fig. 4b).

### MORT is likely an upstream inhibitor of miR-93 in AC16 cells

**Fig. 5:**
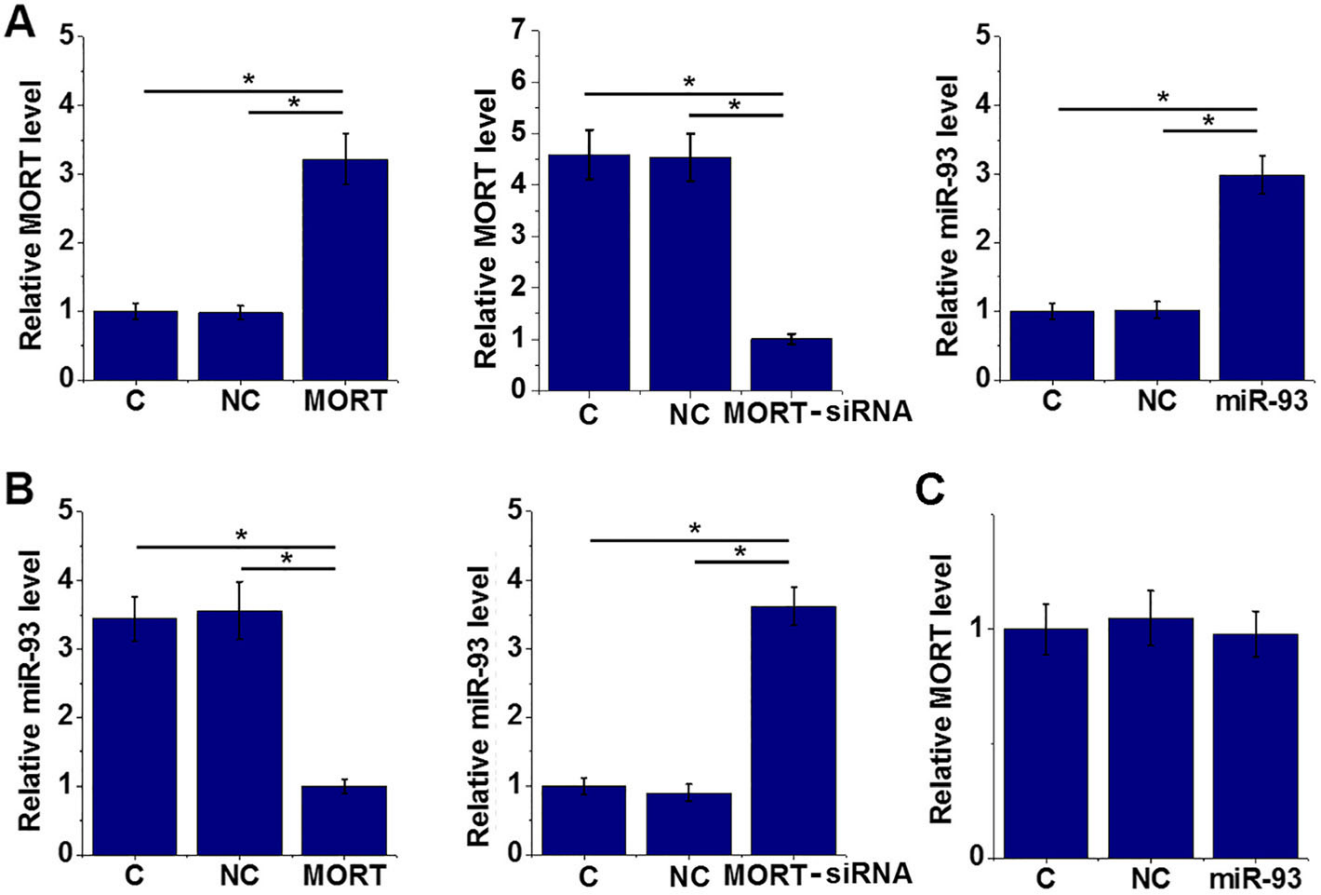
MORT is likely an upstream inhibitor of miR-93 in AC16 cells. AC16 cells were transfected with either MORT expression vector or miR-93 mimic to analyse the interaction between MORT and miR-93. MORT and miR-93 overexpression as well as MORT silencing was achieved in cells of AC16 cell line at 36 h after transfection (**a**). MORT overexpression led to downregulated and MORT siRNA silencing led to upregulated miR-93 expression (**b**), while miR-93 overexpression did not significantly affect MORT expression (**c**) (*, *p* < 0.05)

MORT and miR-93 were overexpressed and MORT was also silenced in cells of AC16 cell line (Fig. 5a). Compared with control (C) and negative control (NC) groups, MORT overexpression led to inhibited and MORT siRNA silencing led to upregulated miR-93 expression (Fig. 5b, *p* < 0.05), while miR-93 overexpression did not significantly affect MORT expression (Fig. 5c).

### MORT promoted the apoptosis the AC16 cell apoptosis by inhibiting miR-93

**Fig. 6:**
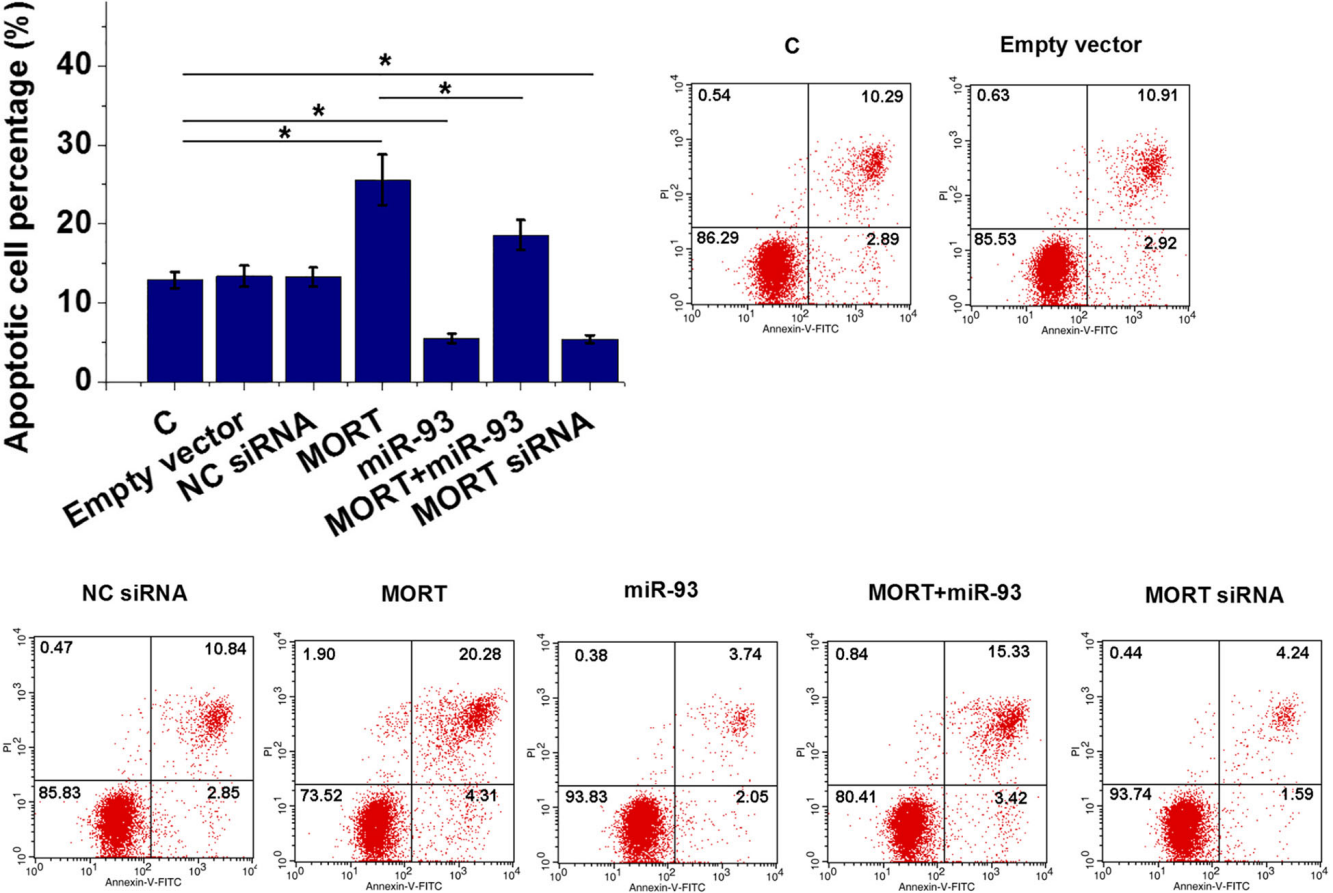
MORT promoted the apoptosis the AC16 cell apoptosis by inhibiting miR-93. The roles of MORT and miR-93 in the apoptosis of AC16 cells were analysed by cell apoptosis assay. MORT overexpression significantly promoted AC16 cell apoptosis, while miR-93 overexpression played and opposite role and attenuated the effects of MORT overexpression. In addition, MORT inhibited the apoptosis of AC16 cells(*, p < 0.05)

Cell apoptosis assay results showed that, compared with control (C) and negative control (NC) groups, MORT overexpression significantly promoted AC16 cell apoptosis, while miR-93 overexpression played and opposite role and attenuated the effects of MORT overexpression. In addition, MORT inhibited the apoptosis of AC16 cells (Fig. 6, *p* < 0.05).

## Discussion

MI is a severe disease causing unacceptable high mortality rate. Therefore, novel therapeutic target is always needed. The key finding of the present study is that lncRNA MORT is upregulated in MI and my promote MI by downregulating miR-93, which has protective effects on cardiomyocyte apoptosis.

Different from protein-coding messenger RNAs, lncRNAs are usually specifically expressed in certain types of tissues [18], indicating the function of lncRNAs in specific biological processes. However, lncRNAs can also enter circulation systems to achieve systemic trafficking [19]. Therefore, lncRNAs may serve as a signal molecule to induce systemic responses under certain disease conditions. In the present study we proved the downregulation of MORT in both heart tissues and plasma of MI model mice compared with Sham mice. Interestingly, plasma levels of MORT were significantly and positively correlated with expression levels of MORT in heart tissues. Those observations suggested that the MORT synthesized in heart tissues can enter blood. Therefore, detecting plasma levels of MORT may reflect its expression in heart tissue and provide guidance for the diagnosis of MI.

LncRNAs participate in human disease through the regulation of downstream genes at multiple levels [5]. For instance, lncRNA CAIF blocks p53-mediated myocardin transcription to suppress heart cell autophagy and attenuate myocardial infarction [20]. However, the interaction between lncRNA and other non-coding RNAs, such as miRNAs, has not been well studied. In our study we showed that MORT was likely an upstream inhibitor of miR-93, which has been reported to have protective effects on cardiomyocyte apoptosis [16]. In addition, the downregulation of miR-93 by MORT is involved in the regulation of cardiomyocyte apoptosis. Therefore, MORT may promote MI by promoting cardiomyocyte apoptosis through the downregulation of miR-93. However, the mechanism of the regulatory effects of MORT on miR-93 is still unclear. It has been reported that lncRNAs may regulate the expression of miRNAs to participate in human diseases [21]. Therefore, MORT may regulate the methylation of miR-93 genes. Future studies may perform luciferase assays to further analyse the interactions between miR-93 and MORT. In vivo anima models are also needed to further study the roles of MORT in MI.

It is worth noting that miR-93 overexpression only partially reduced the enhancing effects of MORT overexpression on cardiomyocyte apoptosis. Therefore, MORT may interact with multiple downstream targets to regulate cardiomyocyte apoptosis. After the establishment of MI model, off-white colour was observed on the apex of the heart and myocardium located on the left ventricle anterior wall. So we think the model was constructed successfully based on the descriptions in a previous study [17]. However, the lack of data confirming successful establishment of the MI model is a limitation of the study. Our future studies will solve this problem.

It is also possible that MORT may sponge miR-93 to play its roles. A miRNA sponge will not affect the expression level of miRNA. It only inhibits the function of miRNA. Preliminary dual luciferase activity assay also showed that MORT and miR-93 could not directly interact with each other (data not shown). Therefore, MORT is unlikely a sponge of miR-93.

## Conclusion

In conclusion, MORT is overexpressed in MI, and MORT may promote MI by promoting cardiomyocyte apoptosis through the downregulation of miR-93.

## Data Availability

The analyzed data sets generated during the study are available from the corresponding author on reasonable request.

## Abbreviations

lncRNAs: Long non-coding RNAs
MI: Myocardial infarction
